# Phenotypic characterization of *Astragalus glycyphyllos* symbionts and their phylogeny based on the 16S rDNA sequences and RFLP of 16S rRNA gene

**DOI:** 10.1007/s10482-014-0163-y

**Published:** 2014-04-08

**Authors:** Sebastian Gnat, Magdalena Wójcik, Sylwia Wdowiak-Wróbel, Michał Kalita, Aneta Ptaszyńska, Wanda Małek

**Affiliations:** 1 Department of Veterinary Microbiology, University of Life Sciences, 12 Akademicka st., 20-033 Lublin, Poland; 2 Department of Botany and Mycology, University of Maria Curie-Skłodowska, 19 Akademicka st., 20-033 Lublin, Poland; 3 Department of Genetics and Microbiology, University of Maria Curie-Skłodowska, 19 Akademicka st., 20-033 Lublin, Poland

**Keywords:** *Astragalus glycyphyllos* symbionts, Phenotypic properties, 16S rDNA sequence analysis, Phylogeny

## Abstract

In this study, the nitrogen fixing *Astragalus glycyphyllos* symbionts were characterized by phenotypic properties, restriction fragment length polymorphism (RFLP), and sequences of 16S rDNA. The generation time of *A. glycyphyllos* rhizobia in yeast extract mannitol medium was in the range 4–6 h. The studied isolates exhibited a low resistance to antibiotics, a moderate tolerance to NaCl, assimilated di- and trisaccharides, and produced acid in medium containing mannitol as a sole carbon source. In the cluster analysis, based on 86 phenotypic properties of *A. glycyphyllos* symbionts and the reference rhizobia, examined isolates and the genus *Mesorhizobium* strains were placed on a single branch, clearly distinct from other lineages of rhizobial genera. By the comparative analysis of 16S rRNA gene sequences and 16S rDNA–RFLP, *A. glycyphyllos* nodulators were also identified as the members of the genus *Mesorhizobium.* On the 16S rDNA sequence phylogram, the representatives of *A. glycyphyllos* nodule isolates formed a robust, monophyletic cluster together with the *Mesorhizobium* species at 16S rDNA sequence similarity of these bacteria between 95 and 99 %. Similarly, the cluster analysis of the combined RFLP–16S rDNA patterns, obtained with seven restriction endonucleases, showed that *A. glycyphyllos* rhizobia are closely related to the genus *Mesorhizobium* bacteria. The taxonomic approaches used in this paper allowed us to classify the studied bacteria into the genus *Mesorhizobium*.

## Introduction

The Fabaceae is one of the largest families of the flowering plants with nearly 19,000 species distributed throughout the world (Allen and Allen 1981). Most of these plants exhibit a unique ability to induce the root and/or stem nodules and establish symbiotic interaction with the soil bacteria called rhizobia. Inside nodules, rhizobia convert nitrogen from atmosphere into ammonium which is assimilated by plant (Haag et al. 2013; Perret et al. 2000). Due to agricultural and ecological importance of N_2_ fixing nodule bacteria, their diversity and taxonomy have been investigated extensively and, in a consequence, the *Rhizobium* systematics has been greatly improved in the last three decades. At the present time, 150 *Rhizobium* species have been identified and assigned to 12 genera of the class alpha-Proteobacteria i.e. *Azorhizobium*, *Bradyrhizobium*, *Mesorhizobium*, *Rhizobium*, *Ensifer*, *Methylobacter*, *Devosia*, *Ochrobactrum*, *Blastobacter*, *Microvirga*, *Shinella*, *and Phyllobacterium* as well as to four genera of the class beta-Proteobacteria i.e. *Burkholderia*, *Cupriavidus*, *Herbaspirillum*, and *Ralstonia* (Balachandar et al. 2007; Małek and Sajnaga 1999; Parte 2014; Sawada et al. 2003; Willems 2006).

Currently, in the *Rhizobium* taxonomy the polyphasic approach, based on phenotypic and genomic criteria is used (Graham et al. 1991; Małek and Sajnaga 1999; Vandamme et al. 1996). Among the phenotypic features especially interesting are those that are important in the ecological niche occupied by a given microorganism. For the classification of bacteria, mainly at the genus level and for tracing their evolutionary history, the analysis of full-length 16S rRNA gene sequences of a few carefully selected strains and restriction fragment length polymorphism (RFLP)–16S rDNA fingerprints of large number of strains are recommended (Mierzwa et al. 2009; Safronova et al. 2014; Smibert and Krieg 1981; Toolarood et al. 2012; Vinuesa et al. 2005; Wdowiak-Wróbel and Małek 2010; Wolde-meskel et al. 2005).

Most taxonomic studies of rhizobia have been focused on the symbionts of the crop legumes of agricultural significance but in the recent years the essential contribution in this field had the investigations of the symbionts of the wild growing legumes which are plants ecologically important.

*Astragalus glycyphyllos*, commonly known as the liquorice milkvetch, is a perennial, herbaceous, leguminous plant widespread throughout Europe and temperate Asia, important to maintain the proper ecosystem functioning. To the best of our knowledge, this is the first report concerning the genus position and evolutionary history of diazotrophic symbionts of *A. glycyphyllos* inferred from numerical analysis of phenotypic properties and comparative sequence analysis of 16S rDNA. In order to determine the genus position and phylogeny of rhizobia associated with *A. glycyphyllos*, we isolated them from liquorice milkvetch root nodules and characterized by series of cultural, physiological, and biochemical tests as well as by sequence and RFLP analyses of the amplified 16S rRNA gene.

## Materials and methods

### Bacterial strains and their maintenance

All strains used in this study are listed in Table 1. Rhizobia were isolated from the root nodules of *A. glycyphyllos* growing in the xerothermic grasslands of south-east part of Poland (Lublin region), although, they could be also found in bulk soil. Nodule bacteria were routinely grown at 28 °C (unless otherwise noted) and stored at 4 °C on yeast extract mannitol medium (YEM 10 g of active dried Baker’s yeast, 10 g of mannitol, 2.5 g of peptone, 15 g of agar, 1 l of deionized water). In phenotypic growth tests on various carbon an nitrogen sources minimal BS medium (Sherwood 1970) was used. Organic substrates were added to the medium after filter sterilization (pore size, 0.22 µm, Millipore Corporation).

**Table 1:** Bacterial strains used in this study

Strains	Host	Geographic origin	Source
*Astragalus glycyphyllos* isolates AG1-29	*Astragalus glycyphyllos*	Poland	ZGM
*Mesorhizobium albiziae* CCBAU61158	*Albizia kalkora*	China	CCBAU
*Mesorhizobium amorphae* ICMP15022	*Amorpha fruticosa*	New Zealand	ICMP
*Mesorhizobium caraganae* CCBAU11299	*Caragana* spp.	China	CCBAU
*Mesorhizobium chacoense* USDA4963	*Prosopis alba*	Argentina	USDA
*Mesorhizobium ciceri* UPMCa7^T^ (ATCC51585^T^)	*Cicer arietinum*	Spain	ATCC
*Mesorhizobium ciceri* USDA3383	*Cicer arietinum*	Spain	USDA
*Mesorhizobium gobiense* CCBAU83330	*Oxytropis glabra*	China	CCBAU
*Mesorhizobium huakuii* USDA4779	*Astragalus sinicus*	China	USDA
*Mesorhizobium loti* USDA3471	*Lotus corniculatus*	New Zealand	USDA
*Mesorhizobium plurifarium* USDA3707	*Acacia senegal*	Senegal	USDA
*Mesorhizobium septentrionale* SDW018	*Astragalus adsurgens*	China	CCBAU
*Mesorhizobium shangrilense* CCBAU65327	*Caragana bicolor*	China	CCBAU
*Mesorhizobium temperatum* LMG23931	*Astragalus adsurgens*	China	LMG
*Mesorhizobium tianshanense* USDA3592	*Glycyrrhiza pallidiflora*	China	USDA
*Bradyrhizobium japonicum*			
USDA110	*Glycine max*	USA	USDA
USDA6	*Glycine max*	USA	USDA
*Bradyrhizobium liaoningense* USDA3622	*Glycine max*	USA	USDA
*Bradyrhizobium* sp. (Lupinus) USDA3045	*Lupinus* sp.	USA	USDA
*Bradyrhizobium yuanmingense* CCBAU10071	*Lespedeza cuneata*	China	CCBAU
*Rhizobium galegae*			
HAMBI1141	*Galega officinalis*	New Zealand	HAMBI
HAMBI1155	*Galega orientalis*		HAMBI
HAMBI1185	*Galega officinalis*		HAMBI
*Rhizobium leguminosarum*			
bv. *trifolii* 21	*Trifolium* sp.	Poland	ZGM
bv. *trifolii* ANU843	*Trifolium* sp.	Poland	ZGM
bv. *viciae* 1	*Vicia* sp.	Poland	ZGM
bv. *viciae* 2	*Vicia* sp.	Poland	ZGM
bv. *viciae* 33	*Vicia* sp.	Poland	ZGM
bv. *viciae* 36	*Vicia* sp.	Poland	ZGM
bv. *viciae* 3841	*Vicia* sp.	Poland	ZGM
*Rhizobium tropici* OUT21	*Phaseolus* sp.	USA	USDA
*Sinorhizobium fredii*			
USDA1-6	*Glycine* sp.	China	USDA
USDA16-1	*Glycine* sp.	China	USDA
USDA440	*Glycine* sp.	China	USDA
*Ensifer meliloti*			
SU47	*Medicago sativa*	Australia	NZP
11	*Medicago sativa*	Poland	ZGM
13	*Medicago sativa*	Poland	ZGM
L5-30	*Medicago sativa*	Poland	ZGM
L54	*Medicago sativa*	Poland	ZGM
MVII	*Medicago sativa*	Poland	ZGM
*Agrobacterium tumefaciens* B6S3		Poland	IChB

*ZGM* Department of Genetics and Microbiology, University of Marie Curie-Skłodowska, Lublin, Poland, *USDA* United States Department of Agriculture, Beltsville, MD, USA, *ATCC* American Type Culture Collection, Rockville, MD, *ICMP* International Collection of Microorganisms from Plants, Landcare Research, Auckland, New Zealand, *LMG* Belgian Coordinated Collections of Microorganisms/LMG Bacteria Collection, Gent Universiteit, Belgium, *CCBAU* Culture Collection of Beijing Agricultural University, Beijing, China, *IChB* Institute of Bioorganic Chemistry, Poznań, Poland, *NZP* Division of Scientific and Industrial Research, Palmerston North, New Zealand, *DB* Department of Biotechnology, Graduate School of Engineering, Osaka University, Yamadaoka, Suitashi, Osaka, Japan

### Isolation of bacteria from *A. glycyphyllos* root nodules

The fresh nodules were dissected from the roots, rinsed thoroughly in water, surface sterilized by immersion in a 0.1 % HgCl_2_ for 1 min, next in 0.75 % ethanol for 1 min, and rinsed in sterile water. Nodules were individually crushed, material from nodules was streaked onto the surface YEM plates, and the plates were incubated at 28 °C for 5–7 days. Individual colonies were selected and their purity was checked by repeated striking of single colonies on YEM medium. The symbiotic ability of each *A. glycyphyllos* nodule isolate was confirmed by nodulating its original host using the method of Vincent (1970).

### Morphological and growth characteristics of bacteria

The nodule bacteria were examined for Gram reaction, structure of colonies, their color, consistency, and presence or absence of gummy substances (Smibert and Krieg 1981). The generation time of *A. glycyphyllos* rhizobia, their growth temperature ranges (5, 12, 15, 20, 28, 35, 40, 42, 45 °C), the ability to grow at various pH (4, 5, 6, 7, 8, 9, 10), tolerance for different NaCl concentrations (0.1, 0.5, 1.0, 1.5, 2.0, 2.5, 3.0 %) were performed on YEM medium as described by Smibert and Krieg (1981). The active movement of bacteria was assessed on 0.3 % YEM agar medium by their capacity to migrate away from the spot of inoculation.

### Utilization of carbon sources

Assimilation of different substrates as a sole carbon sources was tested on BS agar medium (Sherwood 1970) where mannitol was replaced by other carbon compound at the final concentration of 1 % (w/v). The following carbon substrates were tested: l-alanine, l-arabinose, dl-arginine hydrochloride, dl-asparagine, cellobiose, dextrin, dulcitol, fructose, galactose, glycerol, glucose, l-glutamine, hippurate, inulin, sodium malate, xylose, tertrate, lactose, lysine, maltose, mannitol, mannose, raffinose, rhamnose, sucrose, sodium citrate, salicin, starch, trehalose, and tyrosine. The pH of medium was adjusted to 7.0 by using sterile 1 M NaOH or 1 M HCl after the carbon source was added. Bromothymol blue at the final concentration of 0.0025 % (w/v) was used as pH indicator. Changes in the pH values of the media were recorded after 5–7 days of bacterial growth at 28 °C. The BS medium with mannitol was used as the positive control whereas BS medium without any carbon source as negative one.

### Utilization of nitrogen sources

Utilization of different substrates as a sole nitrogen was tested by using the basal BS medium in which NH_4_Cl was replaced by the following amino acids: dl-arginine, dl-glutamine, l-histidine monohydrochloride, l-hydroxyproline, isoleucine, l-methionine, dl-proline, dl-serine, l-tyrosine and NaNO_3_. All nitrogen sources were prepared as a stock solutions and added to the medium to obtain the final concentration of 1 %. The growth of bacteria in BS basal medium served as a positive control.

### Intrinsic antibiotic resistance

Intrinsic antibiotic resistance was tested as described earlier (Kalita and Małek 2004) on YEM agar medium supplemented with one of the following antibiotics at the indicated concentrations (µg ml^−1^): ampicillin 50, 100, 200; neomycin 10, 20, 40, 80; streptomycin 25, 50, 100, 200; tetracycline 5, 10, 20, 40; rifampicin 10, 20, 40, 100.

### Tolerance to dyes

Tolerance to dyes was investigated on YEM agar medium containing the following dyes at the indicated concentrations (%, w/v): auramine 0.0065, 0.013, 0.2; safranin 0.0065, 0.013, 0.2; methyl green 0.0065, 0.013, 0.2; methyl red 0.0065, 0.013, 0.2; crystal violet 0.0065, 0.013, 0.2; acridine orange 0.0065, 0.013, 0.2. The positive control for antibiotic and dye tolerances was the bacterial growth on the basal YEM medium under a standard conditions.

### Miscellaneous other tests

Calcofluor and Congo red absorption were tested in YEM medium as described earlier (Wdowiak-Wróbel and Małek 2000). The precipitation of calcium glycerophosphate was examined according to Hofer’s procedure (1941). Melanin production was studied using the method described by Cubo et al. (1998). The reaction to litmus milk, tests for activity of catalase, β-galactosidase, β-d-glucosidase (aesculinase), nitrate reductase, oxidase, peroxidase, phosphatase, and urease were carried out by the methods of Cowan and Steel (1965) whereas the indole acetic acid synthesis was studied according to method of Minamisawa and Fukai (1991). All phenotypic tests were done in triplicate at least twice.

### Data analysis

In all experiments, the growth of bacteria was recorded after 5–7 days of incubation at 28 °C in triplicate. The results of phenotypic tests were coded using a binary system which was used to estimate the simple matching similarity coefficient (SM) of each strain pair and to generate a similarity matrix (Sneath and Sokal 1973). On the basis of the similarity matrix, clustering analysis was performed by the unweighted pair group method averages algorithm (UPGMA) with NT-SYS software package and results were visualized on the phenogram using NT-SYS software.

### DNA isolation

30 ml of 72 h *Rhizobium* liquid cultures in YEM medium were used to isolate genomic DNA according to the method described by Pitcher et al. (1989). The purity and concentration of DNA samples were assessed with spectrophotometer (Bio-Rad, SmartSpec™3000).

### 16S rRNA gene amplification

Primers fD1 (5′-AGAGTTTGATCCTGGCTCAG-3′) and rD1 (5′-AAGGAGGTGATCCAG CC-3′), corresponding to *Escherichia coli* 16S rRNA gene position 8–27 and 1,524–1,540, respectively were used for PCR amplification of almost full-length 16S rDNA of studied bacteria (Weisburg et al. 1991). Amplification with 50–100 ng of pure genomic DNA was carried out according to ReadyMix™ Taq PCR Reaction Mix procedure (SIGMA-Aldrich) by using the following temperature profiles: initial denaturation at 96 °C for 2 min; 30 cycles of 40 s at 94 °C, 40 s at 52 °C, 1 min at 72 °C; followed by a final extension at 72 °C for 3 min and then a hold at 4 °C. To evaluate the effectiveness of PCR reaction, five microliter portions of amplified DNA were separated electrophoretically on a 1.5 % agarose gel at approximately 80 V. Remaining part of PCR products was purified and sequenced from both strands with the BigDye terminator Cycle Sequencing kit following manufacturer’s instructions (Applied Biosystem, Foster City, CA, USA) and using the 3500 Genetic Analyzer (Applied Biosystems). The amplified 16S rDNA sequences of *A. glycyphyllos* symbionts and those of related bacteria obtained from GenBank were aligned using ClustalW 1.83 package and next, edited with the GeneDoc. Phylogenetic trees were generated using neighbor-joining (NJ) and maximum likelihood (ML) methods. In the NJ method, the phylogenetic distances were determined with Kimura’s two-parameter model (1980) using MEGA 4 program (Tamura et al. 2007). ML analysis was performed using PAUP version 4.0b10 (Swofford 2002) and MODELTEST 3.7 program (Posada and Crondall 1998) to choose the best-fit evolutionary model for 16S rRNA gene. The confidence values for nodes in the tree were generated by bootstrap analysis using 100 permutations of the data sets.

### 16S rDNA nucleotide sequence accession numbers

The 16S rDNA sequences of 10 *A. glycyphyllos* symbionts have been deposited in GenBank nucleotide sequence databases under accession numbers: KF430327–KF430336.

### 16S rDNA PCR-RFLP

Nearly full-length 16S rDNA PCR products, obtained as described above, were digested with 5 U each of *Msp*I, *Mbo*I, *Hin*6I, *Hin*fI, *Taq*I, *Rsa*I, and *Alu*I endonucleases and the restriction fragments were electrophoretically separated in 3 % (w/v) agarose gels. The RFLP profiles obtained with seven enzymes were combined and used for establishing RFLP groups. The isolates that had identical banding patterns were allocated to the same 16S rDNA–RFLP type. Relationships between strains were determined by using data from enzymes that differentiated strains. A binary system (1 for the presence of band and 0 for its absence) was used to generate similarity matrix which was analyzed using the unweighted pair group method with averages algorithm (UPGMA; Sneath and Sokal 1973) and dendrogram, presenting the 16S rDNA–RFLP clusters, was generated from the pairwise similarity matrix with NTSYS-PC software using the Nei and Li (1979) correlation coefficient.

## Results and discussion

In the recent years, the clear progress towards the development of more complete rhizobial taxonomy has been made and new species of nodule bacteria have been described (Balachandar et al. 2007; http://rhizobia.co.nz/taxonomy/rhizobia.html; Sawada et al. 2003; Willems 2006). The present studies, has focused on the analysis of rhizobia isolated from root nodules of wild growing *A. glycyphyllos* in order to determine their genus position and phylogenetic relationship. The International Committee of Systematic Bacteriology recommended minimal standards for valid publication of a new taxa according to which the genus should be built on the numerical analysis of physiological and metabolic features and sequence analysis of 16S rRNA gene whereas, bacterial species should be diagnosable by a discriminative phenotypic properties and DNA–DNA relatedness (Rossellό-Mora and Amann 2001). All studied *A. glycyphyllos* nodule bacteria were effective in nitrogen-fixation with their original host. Effective symbiosis was by the pink color of root nodules and good growth of plants inoculated with bacteria on nitrogen-free medium for 6 weeks (data not presented). Due to a nitrogen-fixing capacity of studied nodule bacteria, their host- *A. glycyphyllos* can colonize nitrogen deficient areas enhancing their fertility and additionally, *A. glycyphyllos*–*Rhizobium* association, potentially is a good candidate for revegetation program.

### Phenotypic characterization and numerical classification of *A. glycyphyllos* nodulators

**Fig. 1:**
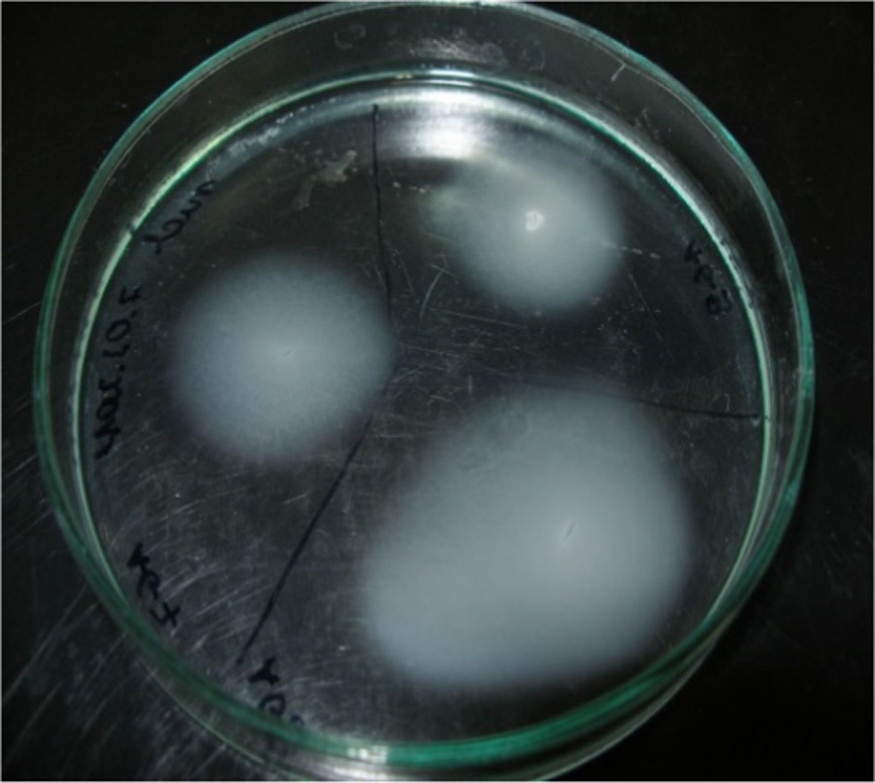
Light microscopy of swarming motility of *A. glycyphyllos* symbionts: AG on 0.35 % swim YEM agar sowing a diffuse swim ring expending beyond the site of bacteria inoculation

A total 62 bacterial strains, including 28 *A. glycyphyllos* nodule isolates and 34 reference strains representing species of the genera: *Rhizobium*, *Ensifer*, *Mesorhizobium*, and *Bradyrhizobium* were analyzed by using 86 phenotypic properties. The phenotypic characterization of *A. glycyphyllos* rhizobia was based on their: generation time, utilization of different compounds as a sole carbon and nitrogen sources, tolerance to NaCl, resistance to different antibiotics and stains, litmus milk reaction, temperature and pH growth range as well as production of some exoenzymes. The liquorice milkvetch rhizobia are Gram-negative, motile bacteria. The bacterial motility was determined on 0.35 % YEM agar medium by flares of growth away from inoculation point (Fig. 1). Temperature is one of the factors affecting the growth of microorganisms. The growth temperature range for *A. glycyphyllos* symbionts was determined between 15 and 34 °C with typical for most rhizobia optimal temperature 28–30 °C (Elkan 1992; Jarvis et al. 1997; Małek and Sajnaga 1999; Mierzwa et al. 2009). All of them grew at pH 6–9. None of tested strains could grow at pH 5 but some of them grew at pH 10. Liquorice milkvetch rhizobia could grow on YEM medium with 0.5 % NaCl and most of them also tolerated 1 % sodium chloride. *A. glycyphyllos* nodulators were moderately fast-growing rhizobia with generation time 4–6 h in YEM broth at 28 °C. Such doubling time is a characteristic of the strains of the genus *Mesorhizobium* (Jarvis et al. 1997; Mierzwa et al. 2009; Van Berkum and Eardly 1998). The strains under investigation were able to use a wide variety of compounds as a sole carbon source. Their carbon utilization patterns varied widely. 14 of the 33 carbon compounds tested supported the growth of all 28 *A. glycyphyllos* nodulators. None of them used sodium hippurate as a sole carbon source. Growing on mannitol and different sugars they acidified medium similar as fast growing rhizobia including mesorhizobia (Elkan 1992). Liquorice milkvetch symbionts assimilated di- and trisaccharides: cellobiose, lactose, maltose, raffinose, and sucrose similar as the reference *Mesorhizobium* species. According to Elkan (1992), this ability is characteristic of fast-growing rhizobia and allow to differentiate them from slow-growing bradyrhizobia. The ability to use a wide range of carbon sources is beneficial for the bacteria living in the soil and may be related to their high competitiveness in a natural environment. Among *A. glycyphyllos* rhizobia some variations in the assimilation of different nitrogen sources were observed. Nine of 22 nitrogen compounds were used by all studied isolates and only l-glycine and dl-isoleucine were not utilized by any of them. The relevant phenotypic characteristics that differentiate newly discovered *A. glycyphyllos* rhizobia from reference *Mesorhizobium* species are listed in Table 2. The growth of *A. glycyphyllos* rhizobia, in the presence of six antibiotics and eight different stains was also tested. These bacteria indicated different patterns for resistance to antibiotics, however, their spectrum of intrinsic antibiotic resistance was relatively narrow. Studied isolates showed a week resistance to tetracycline, neomycin and ampicillin (5–10 µg ml^−1^). They were very susceptible to rifampicin (2.5 µg ml^−1^) and only part of them was resistant to chloramphenicol as well as streptomycin (100 µg ml^−1^). It was found that the susceptibility level of *A. glycyphyllos* nodule isolates to antibiotics was similar to that in fast-growing nodule bacteria of the genera *Mesorhizobium*, *Rhizobium*, and *Ensifer* (Elkan 1992; Garrity et al. 2002; Małek and Sajnaga 1999). For classification and identification of bacteria biochemical tests are also very helpful. They indicated that liquorice milkvetch rhizobia produce acids during the growth in the litmus milk. *A. glycyphyllos* rhizobia showed cytochrome oxidase activity, most of them reduced nitrate to nitrite but any of them could further reduce nitrite to gaseous nitrous oxide. In many strains catalase and urease were expressed but β-galactosidase was not detected.

**Table 2:** Characteristics distinguishing *A. glycyphyllos* symbionts from other *Mesorhizobium* species

Characteristics	*Astragalus glycyphyllos* Nodule isolates	*M. tianshanense* USDA3592	*M. chacoense* USDA4963	*M. mediterraneum* USDA3392	*M. plurifarium* USDA3707	*M. ciceri* USDA3383	*M.huakuii* USDA4779	*M. amorphae* ICMP15022	*M. loti* USDA3471
	*n* ^a^ = 28	*n* = 1	*n* = 1	*n* = 1	*n* = 1	*n* = 1	*n* = 1	*n* = 1	*n* = 1
Tolerance to pH									
4	−^b^	−	−	−	+^b^	−	−	−	−
5	−	−	+	−	+	+	+	+	+
Tolerance to NaCl (%)									
2	+ (9)^c^	−	+	+	+	+	−	+	−
Enzyme activity									
Glucohydrolase-d-glucoside	+ (12)	−	−	+	−	−	+	−	−
Phosphatase	+ (8)	+	+	+	+	+	+	+	+
Nitrate reductase	+ (15)	−	−	−	−	−	−	−	−
Others									
IAA	+ (6)	+	−	−	−	−	+	−	−
Calcofluor, UV	+ (20)	+	+	+	+	−	+	+	+
Nitrogen source									
l-Histidine	+ (23)	+	−	+	−	+	+	−	+
l-Serine	+ (9)	−	+	−	−	−	+	−	+
NH_4_Cl	+ (6)	+	+	+	+	+	+	+	+
dl-Isoleucine	−	−	−	−	−	−	+	−	+
l-Hydroxyproline	+ (13)	+	+	+	+	+	+	+	+
NaNO_3_	+ (12)	−	+	−	+	−	+	+	+
Carbon source									
l-Arginine	+	+	+	+	+	−	+	+	+
l-Lysine	+ (23)	−	+	+	+	−	−	+	+
Salicin	+	−	+	−	+	−	+	+	−
Sodium citrate	+	−	+	−	+	−	+	−	−
Starch	+ (5)	−	−	−	−	+	−	−	+
l-Alanine	+ (7)	−	+	−	−	−	+	+	−
l-Asparagine	+ (20)	−	−	−	+	+	+	+	+
Sodium tartrate	−	−	+	−	+	−	−	+	−
l-Tyrosine	+ (24)	−	−	−	+	−	−	−	+
l-Glutamine	+ (21)	−	−	−	−	−	−	−	−
l-Histidine	+ (7)	−	+	−	−	+	−	+	+
Tolerance to dyes (%)									
Crystal violet									
0.0065	+ (7)	+	+	+	+	+	+	+	+
0.013	−	−	−	+	−	+	+	+	+
Nigrosine									
0.5	+ (21)	−	−	−	+	−	−	+	−
Safranine									
0.05	+ (20)	−	−	+	+	+	+	+	+
Methyl green									
0.0065	−	−	+	+	+	+	+	+	+
0.013	−	−	−	−	+	−	+	−	−
Tolerance to antibiotics (g ml^−1^)									
Neomycin									
10	+ (12)	−	−	−	−	−	+	+	−
Streptomycin									
10	+ (20)	−	−	+	−	+	−	+	−
Rifampicin									
2.5	+ (11)	+	+	+	+	−	+	+	+
10	+ (6)	−	+	−	−	−	+	+	−
Chloramphenicol									
50	+ (20)	−	+	−	−	−	−	+	−
Tetracycline									
10	+ (15)	−	−	−	−	−	−	−	−
Ampicillin									
10	+ (17)	−	−	−	+	−	−	+	−

^a^Number of studied strains

^b^+, − Strains were positive, negative, respectively

^c^Numerical value in parentheses is the number of strains with positive reaction

**Fig. 2:**
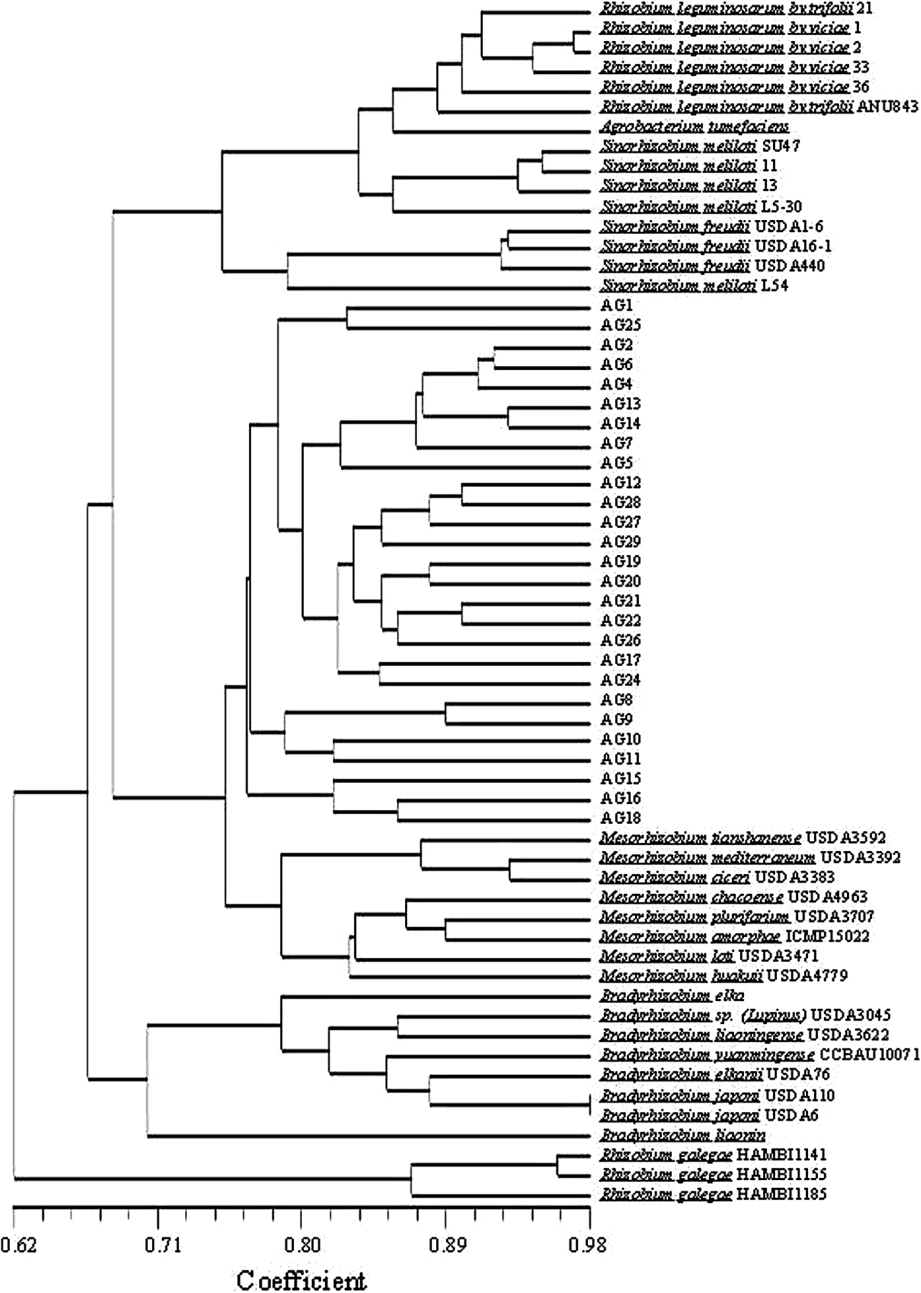
UPGMA dendrogram showing phenotypic relationship between *A. glycyphyllos* rhizobia and reference strains

The phenotypic features, which differentiated strains included into the studies were used for numerical analysis. The results of this cluster analysis, performed by the unweighted pair group method using arithmetic averages, are shown in Fig. 2. All examined nodule bacteria were divided into two major groups at similarity level of 67 % and one independent lineage comprising *R. galegae* strains which branched far from all other tested bacteria. One cluster consisted of fast growing species of the genera: *Ensifer*, *Rhizobium* (except for *R. galegae*), and *Mesorhizobium* as well as *A. glycyphyllos* nodule isolates whereas second one comprised slow-growing bacteria of the genus *Bradyrhizobium*. In the cluster comprising fast-growing rhizobia, all 28 *A. glycyphyllos* nodule isolates formed one large taxonomic subgroup at SM of 0.76. They were found to be closely related to the reference bacteria of the genus *Mesorhizobium* and formed with them one phenon at the similarity level of 75 %. This phenon was clearly separated from cluster comprising *Rhizobium* and *Ensifer* species (Fig. 2). The fact that liquorice milkvetch rhizobia and *Mesorhizobium* species grouped together in one phenon at coefficient similarity of 0.75 preliminary indicates that studied *A. glycyphyllos* nodulators belong to the genus *Mesorhizobium.* We also suggest that large phenotypic diversity of the studied *A. glycyphyllos*–*Rhizobium* population, assessed by numerical analysis, may be essential attribute for its good survival under different environmental conditions and may contribute to the adaptation of these bacteria to the changing soil conditions.

### 16S rDNA sequencing and analysis

**Fig. 3:**
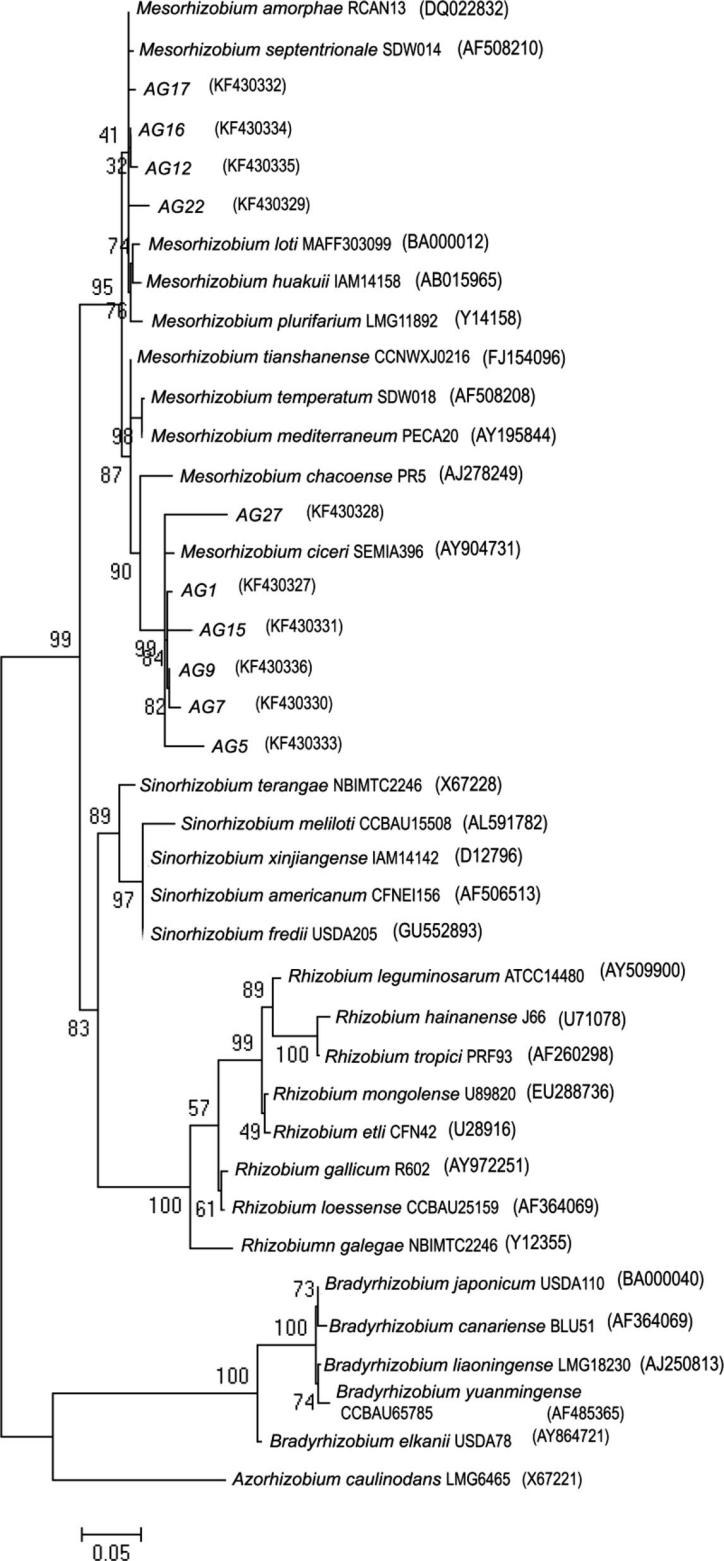
Maximum likelihood tree displaying the phylogenetic relationship of *A. glycyphyllos* symbionts to other rhizobia based on 16S rRNA gene sequences (GenBank accession numbers of 16S rDNA sequences from this study are given in *parenthesis* after the name of *A. glycyphyllos* symbionts)

Analysis of 16S rDNA sequences is widely used to define the taxonomic position and tracing evolutionary history of nodule bacteria (Kalita and Małek 2010; Mierzwa et al. 2010; Shamseldin et al. 2013; Vinay et al. 2013; Vinuesa et al. 2005; Wdowiak-Wróbel and Małek 2010). In order to clarify the taxonomic status and the phylogenetic relationship of *A. glycyphyllos* nodule isolates and other rhizobia, PCR amplification and sequencing of nearly full-length 16S rRNA gene (1,239 bp long) of ten symbionts, representing different phena of liquorice milkvetch rhizobia were performed. 16S rDNA sequences were aligned and compared with those of other rhizobia available in the GenBank database. Evolutionary distances between *A. glycyphyllos* rhizobia and reference bacteria were calculated and molecular phylogeny was reconstructed using the ML and NJ methods (Kimura 1980; Posada and Crondall 1998). The phylogenetic relationships between the studied, yet unclassified rhizobia and the previously named species belonging to the genera: *Mesorhizobium*, *Rhizobium*, *Ensifer*, *Bradyrhizobium*, and *Azorhizobium* is presented only as the ML dendrogram (Fig. 3) since the bacterial clusters strongly supported by the ML distance method were not contradicted by NJ method and vice versa. The results from comparative 16S rDNA sequence studies (Fig. 3) confirmed those from numerical analysis of phenotypic properties (Fig. 2). None of the 16S rDNA sequences of *A. glycyphyllos* nodulators and those of reference rhizobia included into analysis were identical. Their pairwise lowest and highest level of 16S rDNA sequence similarity was 85 and 99 %, respectively. The 16S rDNA sequences of novel isolates also does not perfectly match each other and they were determined to share 95–99 % identical sequences. The meso-growing *A. glycyphyllos* rhizobia were phylogenetically very close to *Mesorhizobium* species with 16S rDNA sequence divergence between 1 and 5 %. It is known that 95 % and higher sequence identities in 16S rDNA sequences serve as “gold standard” for bacterial classification at the genus level (Graham et al. 1991; Madhaiyan and Poonguzhali 2014; Mierzwa et al. 2009; Rosselló-Mora and Amann 2001; Stackebrandt and Goebel 1994; Vandamme et al. 1996; Willems and Collins 1993). Based on the 16S rDNA sequence analysis, *A. glycyphyllos* nodulating rhizobia were assigned to the genus *Mesorhizobium*. The level of nucleotide sequence similarity between the 16S rDNA of *A. glycyphyllos* symbionts and those of *Rhizobium*, *Ensifer*, *Bradyrhizobium* and *Azorhizobium* species were 90–93, 91–95, 85–87 and 87–90 %, respectively. Bacteria of the *Rhizobium*, *Ensifer*, *Bradyrhizobium* and *Azorhizobium* genera formed on the 16S rDNA phylogram separate, monophyletic lineages with *Bradyrhizobium* species and *Azorhizobium caulinodans* as an outliers (Fig. 3).

On the 16S rDNA phylogram, all novel rhizobia clustered together with the *Mesorhizobium* species with high confidence (95 % bootstrap) and they were divided into two subgroups (Fig. 3). One of them consisted of *Mesorhizobium tianshanense* CCNWXJ0216, *Mesorhizobium temperatum* SDW018, *Mesorhizobium mediterraneum* PECA20, *Mesorhizobium chacoense* PR5, *Mesorhizobium ciceri* SEMIA396, and *A. glycyphyllos* nodulators: AG1, AG5, AG7, AG9, AG15, AG27. The second subgroup comprised: *Mesorhizobium amorphae* RCAN13, *Mesorhizobium septentrionale* SDW014, *Mesorhizobium loti* MAFF303099, *Mesorhizobium huakuii* IAM14158, *Mesorhizobium plurifarium* LMG11892 as well as liquorice milkvetch symbionts: AG12, AG16, AG17, and AG22 (Fig. 3).

**Table 3:** RFLP analysis of PCR-amplified 16S rDNA of *A. glycyphyllos* isolates and reference mesorhizobial strains used in this study

Strains	Restriction pattern types^a^ of amplified 16S rDNAs digested with:							16S rDNA–RFLP genotypes^b^	16S rDNA–RFLP patterns groups^c^
	*Alu*I	*Mbo*I	*Rsa*I	*Hin*6I	*Taq*I	*Msp*I	*Hin*fI		
*AG*1	A	A	A	A	A	B	A	AAAAABA	I
*AG*2	A	A	A	C	A	B	A	AAACABA	II
*AG*3	A	A	A	C	A	B	A	AAACABA	II
*AG*4	A	A	A	A	C	B	A	AAAACBA	III
*AG*5	A	A	A	A	A	B	A	AAAAABA	I
*AG*6	A	A	A	A	A	B	A	AAAAABA	I
*AG*7	A	A	A	A	A	B	A	AAAAABA	I
*AG*8	A	A	A	C	A	B	A	AAACABA	I
*AG*9	A	A	A	C	A	B	A	AAACABA	I
*AG*10	A	A	A	C	B	B	A	AAACBBA	IV
*AG*11	A	A	A	A	B	B	A	AAAABBA	V
*AG*12	A	A	B	A	A	A	E	AABAAAE	VI
*AG*13	A	A	A	B	A	D	B	AAABADB	VII
*AG*14	A	A	A	B	A	D	B	AAABADB	VII
*AG*15	A	A	A	B	A	D	B	AAABADB	VII
*AG*16	A	A	A	A	C	A	B	AAAACAB	VIII
*AG*17	A	A	A	A	A	A	B	AAAAAAB	IX
*AG*18	A	A	B	A	A	A	B	AABAAAB	X
*AG*19	A	A	B	C	A	A	B	AABCAAB	XI
*AG*20	A	A	A	A	A	C	A	AAAAACA	XII
*AG*21	A	A	B	A	A	C	A	AABAACA	XIII
*AG*22	A	A	B	A	A	A	B	AABAAAB	X
*AG*24	A	A	B	A	A	C	A	AABAACA	XIII
*AG*25	A	A	B	A	B	C	A	AABABCA	XIV
*AG*26	A	A	B	B	A	D	B	AABBADB	XV
*AG*27	A	A	B	A	B	C	A	AABABCA	XIV
*AG*28	A	A	B	A	A	C	A	AABAACA	XIII
*AG*29	A	A	B	A	A	C	A	AABAACA	XIII
*Mesorhizobium albiziae* CCBAU61158	A	B	C	B	D	E	F	ABCBDEF	XVI
*Mesorhizobium amorphae* ICMP15022	A	A	A	B	A	A	B	AAABAAB	XVII
*Mesorhizobium caraganae* CCBAU11299	A	A	A	A	A	C	C	AAAAACC	XVIII
*Mesorhizobium ciceri* USDA3383	A	A	A	A	A	D	C	AAAAADC	XIX
*Mesorhizobium chacoense* USDA4963	A	A	A	D	A	F	B	AAADAFB	XX
*Mesorhizobium gobiense* CCBAU83330	A	A	D	A	A	G	H	AADAAGH	XXI
*Mesorhizobium huakuii* USDA4779	A	A	A	B	A	A	B	AAABAAB	XVII
*Mesorhizobium plurifarium* USDA3707	A	A	A	A	E	A	D	AAAAEAD	XXII
*Mesorhizobium loti* MAFF303099	A	A	A	B	A	A	D	AAABAAD	XXIII
*Mesorhizobium septentrionale* SDW018	A	A	A	A	A	A	B	AAAAAAB	IX
*Mesorhizobium shangrilense* CCBAU65327	A	A	A	A	A	A	F	AAAAAAF	XXIV
*Mesorhizobium tianshanense* USDA3592	A	A	A	A	A	C	A	AAAAACA	XII
*Mesorhizobium temperatum* LMG23931	A	A	A	B	A	C	C	AAABACC	XXV

^a^Letters (A–G) refer to 16S rDNA–RFLP pattern types of studied rhizobia detected with each used restriction enzyme

^b^Combinations of letters (A–G) refer to 16S rDNA genotypes of studied rhizobia detected by the combined RFLP analyses of 16S rDNA digested with *Msp*I, *Mbo*I, *Hin*6I, *Hin*fI, *Taq*I, *Rsa*I, and *Alu*I endonucleases

^c^Roman numerals refer to 16S rDNA–RFLP pattern groups of studied rhizobia identified by the combined analyses of 16S rDNA restriction profiles obtained with seven endonucleases used

### RFLP analysis of PCR amplified 16S RNA genes

RFLP analysis of 16S rDNA is recognized as a powerful and rapid method to determine the phylogenetic relationship of large number of legume root nodule isolates (Kalita and Małek 2010; Mierzwa et al. 2010; Shamseldin et al. 2013; Sikora and Redžepović 2003; Vinay et al. 2013; Vinuesa et al. 2005; Wolde-meskel et al. 2005). To elucidate the phylogeny and genus position of all 28 *A. glycyphyllos* nodule isolates, fingerprinting of 16S rDNA by RFLP has been used. The 16S rDNAs of liquorice milkvetch rhizobia as well as those of reference bacteria of the genus *Mesorhizobium* were amplified via PCR with universal primers fD1 and rD1 (Weisburg et al. 1991). Amplification provided for each studied strain a single band of about 1,5 kb long. The PCR products were digested separately by seven 4-base cutting enzymes and the resulting fragments were separated by electrophoresis (data not presented). Two enzymes (*Alu*I and *Mbo*I) did not established different RFLP patterns for *A. glycyphyllos* nodule isolates and produced the same 16S rDNA profile for all of them. Remaining five endonucleases (*Msp*I, *Taq*I, *Hin*6I, *Rsa*I, *Hin*fI) differentiated studied isolates and established specific to them 16S rDNA–RFLP patterns. *Alu*I produced identical DNA profiles for *A. glycyphyllos* symbionts and reference rhizobia, whereas *Hin*fI was the most discriminating restriction enzyme for these bacteria and was able to differentiate among them eight restriction pattern types. The remaining enzymes produced the following number of profiles: 5, *Taq*I; 4, *Hin*6I; 8, *Rsa*I. The 16S rDNA–RFLP patterns of *A. glycyphyllos* nodule isolates were compared with those of the genus *Mesorhizobium* reference strains. The 16S rDNA–RFLP profiles of studied rhizobia, obtained in two independent experiments, were very similar (data not presented). Based on the combined RFLP patterns of 16S rRNA gene, 25 distinct 16S rDNA genotypes were distinguished within all 41 analyzed strains (13 among 28 isolates collected from *A. glycyphyllos* nodules and 12 among 13 reference *Mesorhizobium* species) (Table 3). Our results supported previous reports on heterogeneous nature of the genus *Mesorhiozobium* strains (Mierzwa et al. 2009; Wang et al. 1999, 2007; Wdowiak-Wróbel and Małek 2010). Among studied *A. glycyphyllos* symbionts, the most widespread was genotype designated as: I which comprised six strains. Five other genotypes i.e. XIII, VII, II, X, and XIV occurred less frequently in liquorice milkvetch *Rhizobium* population and were represented by four, three, two, two, and two strains, respectively. Nine genotypes were specific to single *A. glycyphyllos* nodulators. Two of them, 16S rDNA genomic types IX (strain AG17) and XII (strain AG20) showed the 16S rDNA restriction patterns identical to those of *M. septentrionale* SDW018 and *M.* *tianshanense* USDA3592, respectively (Table 3).

**Fig. 4:**
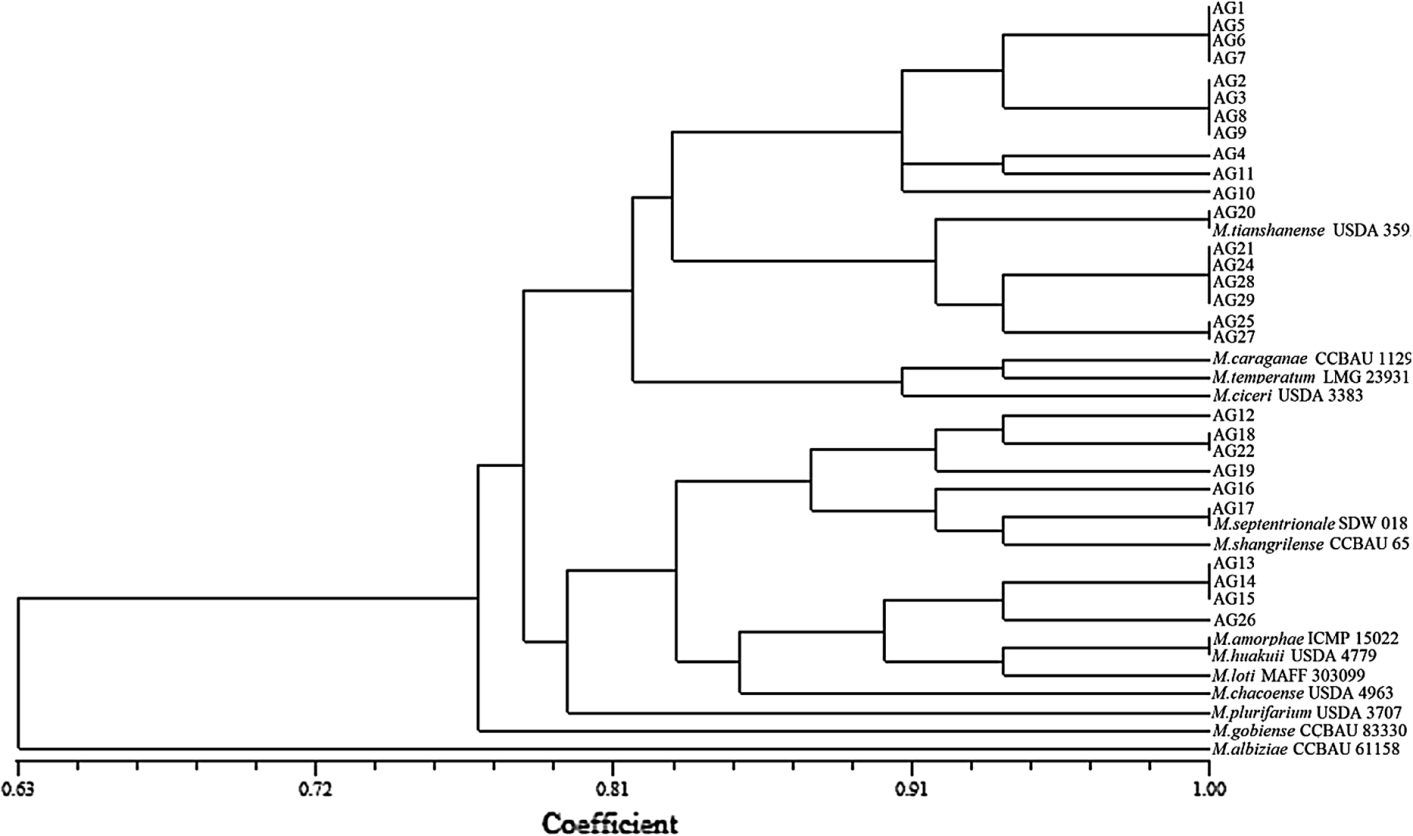
Dendrogram based on the UPGMA cluster analysis of RFLP patterns of 16S rDNA showing phylogenetic relationship of *A. glycyphyllos* symbionts and reference mesorhizobia

The fingerprints of bacterial strains generated by RFLP of 16S rRNA genes were used for a cluster analysis and construction of the dendrogram according to the genetic distance matrix by the UPGMA algorithm. The close phylogenetic relationship of *A. glycyphyllos* symbionts and the genus *Mesorhizobium* bacteria is presented on the 16S rDNA–RFLP phylogram (Fig. 4). All *A. glycyphyllos* nodulators clustered together with *Mesorhizobium* species forming two coherent subgroups at 16S rDNA–RFLP pattern similarities of ~82–83 %. Only *M. gobiense* and *M. albiziae* branched off from the remaining bacteria included into analysis and formed a separate phylogenetic lineages with 16S rDNA–RFLP similarity patterns to them of 77 and 63 %, respectively (Fig. 4). Results obtained in RFLP analysis of 16S rDNA correlated well with those of 16S rDNA sequence analysis. The composition of *A. glycyphyllos* nodulators within two main clusters on the phylograms constructed by 16S rDNA sequences (Fig. 3) and RFLP analyses (Fig. 4) was identical (with exception of AG15 strain). These data led us to conclusion that combine analysis of 16S rDNA–RFLP patterns is a powerful approach for rapid typing and classification of large number of legume root nodule isolates at the genus level.

In conclusion, in the present study by using numerical analysis of phenotypic properties, RFLP and sequence analyses of 16S rDNA we showed that *A. glycyphyllos* nodule bacteria were grouped within the genus *Mesorhizobium* cluster lending strong support to classify the liquorice milkvetch rhizobia in the genus *Mesorhizobium*.
